# The Homœopathic Prescription over the Pathological One

**Published:** 1893-03

**Authors:** Horace P. Holmes

**Affiliations:** Omaha, Neb.


### THE HOMŒOPATHIC PRESCRIPTION OVER THE PATHOLOGICAL ONE.

HORACE P. HOLMES, M. D., OMAHA, NEB.

In this day and age of progress when the acme of medical ability seems to be for scientific innovations, the thought must come to us as to the relative value of the correctly made homœopathic prescription where given according to the peculiar, prominent, uncommon symptoms over the one made on some supposed pathological basis. It is a common occurrence for the writer to be criticized for his belief that the prescription is not best made on pathological symptoms, and that in reality we ignore the best known pathological conditions in making up our most successful prescriptions. That the writer has the correct view of the matter remains unquestioned in his own mind, for the conclusion has been arrived at only after years of patient investigation and the most serious thought he has been able to give the subject. If there is a better way, its demonstration will be hailed with joy and its immediate adoption promised.

Hahnemann taught us that the common symptoms of diseased conditions were of little importance in making up the drug picture of the remedy for the disease that would do the most effective work. At first thought this truly seems an unreasonable statement. That we should throw out such symptoms as pain, fever, chill, inflammation, suppuration, tumors, and all the common features of diseased conditions as practically worthless, and take up those which were peculiar to the individual, and perhaps having no reasonable bearing upon the case from a pathological standpoint seems indeed an unwarrantable position for one to hold. But years of homœopathic practice from this Hahnemannian stand-point has proved the truth of the statement, and we do not lack for ample evidence to substantiate the position.

As a first argument in evidence of the verity of our Hahnemannian position, let me state that we know very little of the pathological conditions of the great majority of affections we are called upon to treat. What is the pathology of rheumatism, and in what possible way can the pathological condition of the case explain why Bryonia is indicated where the patient is worse from movement and Rhus-tox. relieves the patient when better from motion? What is the pathology of diabetes, and in what way can the indication of Apis where there is an absence of thirst be explained on a pathological reasoning? What is the pathology of sick headache, and in what way can the peculiar symptom of Sanguinaria be explained where the patient for relief kneels down and presses the head against the floor? Or what the diseased condition that makes the Silicea patient better from wrapping up warmly, and the Calcarea-carb. patient want cold applications made to the aching head? Given a case of bronchitis, what are you going to give for it from a pathological stand-point? Here is a patient that coughs her head nearly off on coming into a warm room, and another is equally bad on going into a cold atmosphere. Both remain comfortable in the opposite temperature. On what line of pathological reasoning will Bryonia help the one and Phosphorus the other? I was called a few days ago to take charge of a patient already prescribed for by a homœopathic physician. The pathological condition indicated pleurisy of so violent a type that the patient could neither draw a long breath nor lie down. Bryonia had been given with no beneficial result. There was one peculiar symptom, the patient could not lie on the affected side. A dose of Belladonna$^{30}$ was given, and the patient, not only laid down, but turned on the affected side and slept the sweet sleep of the suffering relieved from pain. Why was this so, and how can it be explained on any of the scientific, pathological understandings? It is sufficient for us to know that it *is* so, and that it can be done in almost every instance.

I was called not long since to a very poor family where the county physician had already lost a boy of ten months, and the only remaining boy—a child of three years—was *in extremis* from what scientific practice had diagnosed as follows: "Bronchitis of the lungs, catarrh of the stomach, and inflammation of the bowels." A little allowance for the wording of the diagnosis was made for the physician, as it had come through the family as informant. But unscientific Homœopathy could take no exception to the opinion. Furthermore, the child was very sick, and to all appearances would not survive another twenty-four hours. I refused the case, the father pleaded; I said I thought there was no hope, and that I was a stranger to the family, consequently they could have no confidence in me. The parents felt their only boy was dying, and begged me to make one trial and do what I could. You all know what it is to undertake the treatment of a case that to all appearance must die before the next day is spent. The symptoms pointed to Arsenicum. There was great pallor, great exhaustion, craving for cold water often and it was immediately vomited, frequent diarrhœic stools almost as black as ink, and a restlessness that made it almost impossible to keep the child covered. And the result? Thanks to the inestimable worth of Hahnemann's legacy the boy is to-day running the streets of his neighborhood as well as any little ragamuffin in my parish. And now, my would-be scientific homœopath, what was the pathology of this case that made me give Arsenicum as the homœopathic remedy instead of Belladonna, which is so rationally indicated in many of the same kind of diseased conditions? I cannot tell. I only know that there is enough proof of the efficacy of the method to make me perfectly satisfied to try it again.

Last year I was called one hundred miles to prescribe for a patient for a urinary trouble. I will not give the diagnosis or the pathology for fear of prejudicing the mind of the hearer before he learns the result of the treatment. The patient had suffered for years, and his life had grown to be in jeopardy from the seriousness of his condition. He was a homœopath, and had been prescribed for by some of our leading representatives. While examining his case, the patient said he must get out of bed and urinate. He sank on his knees on the floor with the vessel between his knees. He began to strain in the effort to start the water. Slowly he leaned forward, the face reddening more and more, and the drops of perspiration starting from his forehead in the agony of his suffering. Farther and farther he leaned forward until his forehead touched the floor, and the most agonizing moans and screams were wrung from the old soldier as a few drops of bloody urine dribbled from the urethra. Laying aside all pathological reasonings in this case, I thought of Pareira-brava—a remedy I had often wondered at from the peculiar symptom but had never given—and the remedy certainly did wonders in relieving the case. It is of no importance that the remedy did not establish a perfect cure and that surgical means were resorted to. But a sound could not be passed at the time in spite of every manœuvre known to the writer, and the remedy did what no other remedy had done up to that time.

Another case that attracted my attention to this same line of thought was that of a woman who had gone through an abortion at the sixth week. I had been called to see another member of the family and the mother wished me to prescribe for her. She had gone through the abortion all right, as she supposed, but it left her sore and sensitive across the abdomen. She would feel pretty well when she arose in the morning, but by night she would be sore as to be almost unable to move. "And," she said, "I am so afraid of getting hurt. If my little girl only starts toward me, I am afraid she will hurt me, and I put my hands over my abdomen to protect me." What has Arnica to do with such a mental condition when we consider the pathological features of the case? And yet one prescription of Arnica was all the work I needed to do for the patient. My student, who witnessed the work, and learned of the result, said: "It beats H——!" And after nearly twelve years of experience I still am compelled to as forcibly express myself every few days.

And now, my pathological brethren, I have given the reasons for the faith that is in me, and illustrated why I am compelled to think so. Because I take such a position from honest conviction is the writer necessarily a crank? It is within the possibilities that an exceptional case may be shown where a malady was relieved or cured in which the peculiar, prominent, uncommon symptoms were ignored and that the patient was treated entirely on a scientific basis. Admitting this, it will be very easy to point out a similar case where the scientific methods failed and the strict homœopathic prescription cured the case. *Sic Semper Homœopathy!*

